# The Effects of Intact Cereal Grain Fibers, Including Wheat Bran on the Gut Microbiota Composition of Healthy Adults: A Systematic Review

**DOI:** 10.3389/fnut.2019.00033

**Published:** 2019-03-29

**Authors:** Angie Jefferson, Katie Adolphus

**Affiliations:** ^1^Independent Researcher, Bracknell, United Kingdom; ^2^The Kellogg Company, Manchester, United Kingdom

**Keywords:** cereal fiber, dietary fiber, gut microbiome, gut microbiota, prebiotic, wheat fiber, wheat bran, systematic review

## Abstract

The human microbiota is increasingly recognized as a major factor influencing health and well-being, with potential benefits as diverse as improved immunity, reduced risk of obesity, Type 2 diabetes, and improved cognition and mood. Bacteria inhabiting the gut are dependent on the provision of fermentable dietary substrates making diet a major factor driving the composition of the human gut microbiota. Dietary fiber may modify microbiota abundance, diversity, and metabolism including short-chain fatty acid production. The majority of research to date has explored isolated fibers, and the influence of habitual fiber consumption is less well-established. The aim of the current article was to systematically review evidence from human intervention studies for the effects of intact cereal fibers, and their active sub-fractions, on gut microbiota composition in healthy adults. Studies published in the past 20 years were identified through the PubMed and Cochrane electronic databases. Inclusion criteria were: healthy adult participants (>18 years), inclusion of at least one intact cereal fiber, or its sub-fraction, and measurement of fecal microbiota related outcomes. As every individual has a unique microbiota many trials utilized a cross-over design where individuals acted as their own control. Outcome measures included change to the microbiota, species diversity, or species abundance, or metabolic indicators of microbiota fermentation such as short chain fatty acids or fecal nitrogen. Two hundred and twenty three publications were identified and 40 included in the final review. In discussing the findings, particular attention has been paid to the effects of wheat fiber, bran, and arabinoxylans (AXOS) as this is the dominant source of fiber in many Western countries. Thirty-nine of the forty-two studies demonstrated an increase in microbiota diversity and/or abundance following intact cereal fiber consumption, with effects apparent from 24 h to 52 weeks. Increases in wheat fiber as low as 6–8 g were sufficient to generate significant effects. Study duration ranged from 1 day to 12 weeks, with a single study over 1 year, and exploration of the stability of the microbiota following long-term dietary change is required. Increasing cereal fiber consumption should be encouraged for overall good health and for gut microbiota diversity.

## Introduction

Increased consumption of wholegrains is recommended across the world due to their association with a reduced risk of cardiovascular disease, overweight/obesity, Type 2 diabetes, and cancer (1, 2). One of the proposed mechanisms behind these protective effects is the fermentation of prebiotic cereal dietary fibers by the colonic microbiota (3). The key metabolic outputs of the gut microbiota are short chain fatty acids (SCFA): principally acetate; propionate, and butyrate; each of which effects host health. Acetate is absorbed and metabolized by the brain, muscle, and body tissues, propionate is cleared by the liver and may lower hepatic production of cholesterol, and butyrate provides an energy source to the cells lining the colon and may help to protect against colon disorders (3, 4). In addition, these SCFA also exert anti-inflammatory effects, and are thought to play a role in the modulation of glucose and lipid metabolism (3, 4).

The composition of the gut microbiota has been shown to respond to dietary change, determined by competition for substrates, and by tolerance of the gut conditions (3). However, the majority of research into the beneficial effects of prebiotic fibers has focused on isolated prebiotic fibers, and research into the impact of intact cereal fibers is less well developed. This may be due in part to the complexity of fiber molecules resulting in a high degree of variability in fermentation of different fibers by the colonic microbiota, and also the high variability in baseline gut microbiota between individual participants, adding complexity into any research into intact cereals fibers (5). A summary of the key bacterial phylotypes and the typical variation of phylotype proportions reported in the microbiota of Western populations is provided in Table 1. Evidence is emerging supporting the role of habitual diet in modulating the structure of both the gut microbial composition and also its metabolism (3, 6).

**Table 1:** Major Bacterial Groups in the Human Gut Microbiota and the Main Fermentation Products.

**Major phyla**	**Important families in gut microbiota**	**Main fermentation substrates & by-products**	**Additional notes:**
Firmicutes *Spore forming bacteria—include helpful species* e.g*., lactobacillus and pathogenic* e.g., *clostridium* typically form 64–78% western human microbiota	Lactobacillaceae, Clostridiaceae, Enterococcus, Lachnospiraceae, Roseburia, Dialister, Eubacteriaceae, Ruminococcacea, Anaerostipes,	Carbohydrates—producing SCFA's-acetate, formate, lactate, butyrate, succinate, and propionate Proteins—producing BCFAs, indoles, sulfides, phenols, amines, NH3, H2, CO2, CH4	Diverse group of both beneficial and pathogenic bacteria Contain a number of bacterial species adapted for utilizing fiber as a substrate and producing butyrate Wide range of positive benefits including reduce risk of obesity and Type 2 diabetes, improved glucose tolerance, protection against colon cancer
Bacteroidetes *Opportunistic pathogens* Typically form 13–28% of human gut microbiota in western populations	Bacteroides, Prevotellae, Barnesiella,	Able to metabolize both carbohydrates and proteins. Important fermenters of dietary fiber	Bacteroides dominant in western populations with higher protein and fat intakes. Prevotellae dominate in those consuming high levels of fiber.
Actinobacteria *Producers of metabolites & antibacterial/virals* Typically form 3–5% of human gut microbiota in western populations	Bifidobacteriaceae, Corynebacteriaceae, Atopobium, Eggerthella, Collinsella	Able to breakdown simple sugars, cellulose and hemicellulose Producing lactate, acetate, and formate from carbohydrates	Wide range activities including inhibiting growth pathogens, improvement gut mucosal barrier and vitamin production
Proteobacteria *Gram negative bacteria including a wide range of pathogens* Typically no more than 3% of human gut microbiota in western populations	Enterobacteraceae, Pseudomonadaceae, Helicobacteraceae	Able to ferment carbohydrates and proteins producing lactate, acetate, succinate, and formate from carbohydrates, sulfide from sulfate, H2S, mercaptans from protein	Include a wide range of pathogens

For the purposes of this review, intact cereal fiber included both the soluble and insoluble non-digestible carbohydrates found in cereal grains. To our knowledge, previous systematic reviews (such as 7–9) in this area have included a range of prebiotic fibers largely from supplemented, isolated fiber types (7, 8), or have explored specific conditions such as IBS (9), and there has not been a systematic review exploring the impact of intact cereal fibers consumed in everyday foods (such as breakfast cereals and breads) on the gut microbiota in healthy participants. Therefore, the aim of the current review was to systematically review the evidence from human intervention studies for the effects of intact cereal fibers and their active sub-fractions on gut microbiota composition in healthy adults. A subsidiary aim of the review was to systematically review the effects of wheat bran fiber on gut microbiota composition in healthy adults, as wheat bran fiber is the largest contributor to cereal fiber intake in Western societies. Wheat bran contains high levels of the hemicellulose arabinoxylan, which can be utilized by the bacteria inhabiting the microbiota. As the biggest component of fiber across the Western world, studies examining the role of wheat arabinoxylan and arabinoxylan-oligosaccharides (AXOS) have also been included.

As this review has not been carried out previously, studies published over the past 20 years were included. The effects of intact cereal fibers were evaluated by change in microbiota abundance, diversity, increase in specific bacterial species, plus indicators of microbiota fermentation activity such as short chain fatty acid production and fecal nitrogen.

The current systematic review increases understanding of the impact of consumption of intact cereals fibers on the gut microbiota and consequent health.

## Methods

### Search Strategy and Search Terms

**Figure 1:**
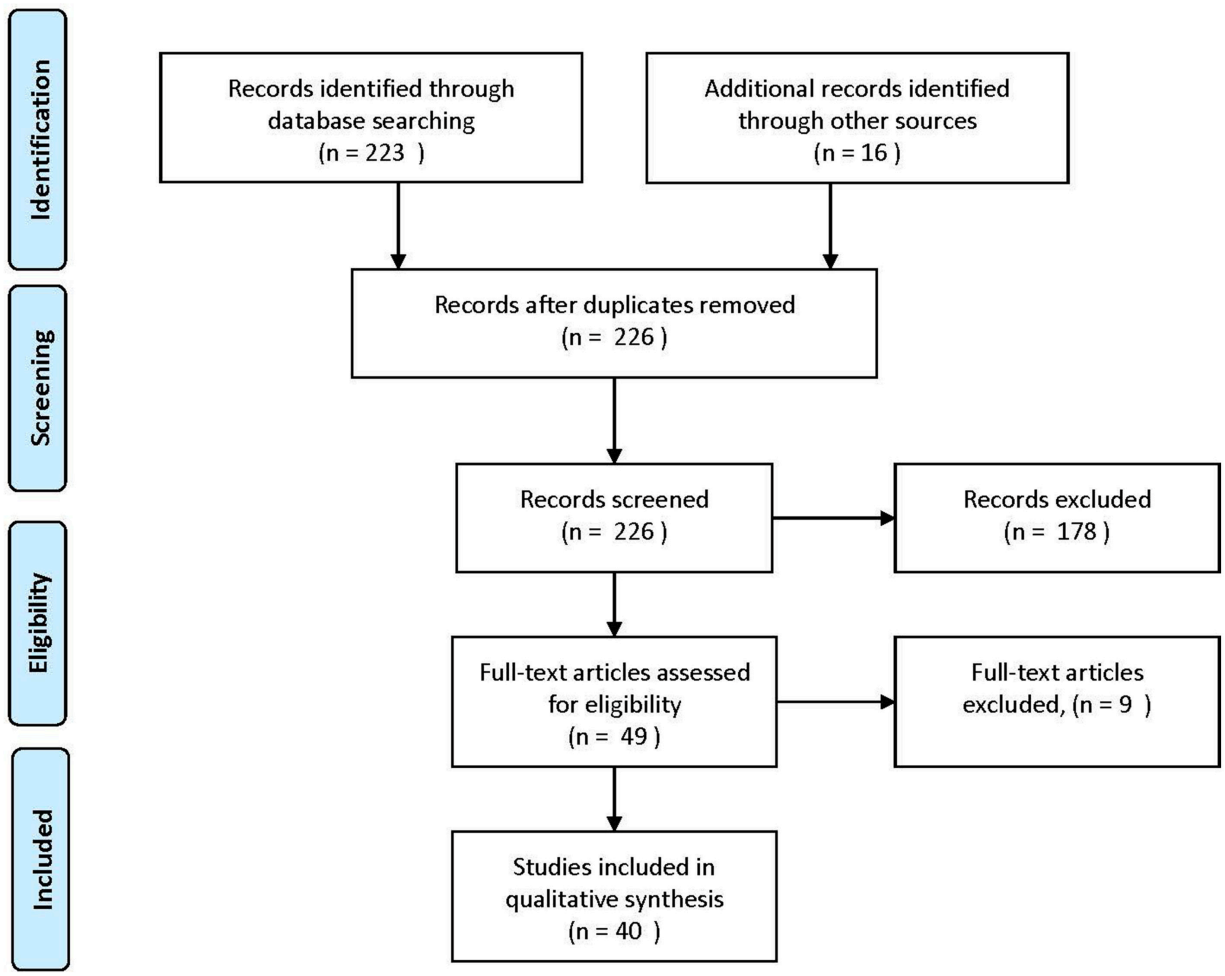
Process of selecting included human studies. Adapted from Moher et al. (10).

The review was reported using Preferred Reporting Items for Systematic Reviews and Meta-Analyses (PRISMA) guidelines. Studies were identified by searching the PubMed and Cochrane electronic database from January 1998 to June 2018. A combination of search terms relevant to this review were used including “microbiome,” “microbiota,” or “prebiotic” in combination with “grain fiber or fiber” “digestive health,” “wheat,” “oat,” “barley,” “rye,” or “rice.” The active sub-fractions of wheat: “arabinoxylans” and “AXOS,” were also included into the search. The reference lists of the studies identified were examined individually to supplement the electronic search. Additional unpublished data was provided by one author to supplement published material (Neacsu M). The process for selecting studies for inclusion in this review is detailed in Figure 1. A total of 42 studies were included in the final review.

### Inclusion and Exclusion Criteria

Publications written in the English language were selected for review. Only original research studies reporting the influence of manipulating intake of an intact cereal fiber via a food based intervention or one of it sub-fractions on the gut microbiota or bacterial fermentation metabolites in human adult (>18 years) participants were included. All experimental designs were eligible for inclusion. Studies that included only participants with a chronic health condition (e.g., Irritable Bowel Syndrome, Ulcerative Colitis etc.) were excluded from this review. Studies that included overweight and obese participants who were otherwise healthy and without abnormal clinical parameters (e.g., elevated blood pressure) were included. Additional detail on potential confounders (such as use of antibiotics, pre/probiotics, and laxative use), was extracted wherever possible, however studies failing to provide this specific detail (*n* = 5) were not excluded in order to capture the microbiota response of free living individuals in the community. There was no restriction on length of fiber manipulation, or mechanism by which it was administered. Studies that included any outcome of objectively measured gut microbiota composition were included. Studies that used the following outcomes measures were included in the review: change to the microbiota in terms of total populations or individual species, or measurement of the metabolites arising from bacterial fermentation such as the short chain fatty acids (acetate, butyrate, and propionate), breath hydrogen or fecal nitrogen. Where significant changes to other parameters were reported (e.g., blood lipids, bowel habit or glucose, and insulin response) these have also been noted in Table 2.

**Table 2:** Summary of human studies exploring the impact of intact cereal fibers on the gut microbiota.

**References**	**Country, recruitment, sex, baseline age (years)**	**Adjustment for confounding variables**	**Fiber source**	**Design**	**Bacterial determination**	**Test period**	**Intervention daily intake**	**Changes to bacterial species**	**Other outcomes**	**Conclusions**
**WHEAT/WHEAT BRAN**										
Costabile et al. (11)	UK 31 adults (15 M & 16 F) Av age 32 y BMI 20–30	Prebiotics, probiotics, high bran or whole grain breakfast cereals, GI drugs, antibiotics (6 m), laxatives, substance or alcohol abuse, major illness, GI disease	Wholegrain wheat breakfast cereal vs. Wheat bran breakfast cereal	Randomized crossover	FISH	3 weeks Washout 2 weeks	① 48 g WG wheat breakfast cereal (5.7 g fiber/serve) ② 48 g wheat bran breakfast cereal (13 g fiber/serve)	① & ② sig ↑ in bifidobacteria, lactobacilli, enteroccoci & aptobiumHowever, ↑'s were sig greater with WG compared to WB cerealSig ↑ clostridium with ②	① & ② sig↑ plasma ferulic acid with both cereals but greater increase with WB WB sig ↑ stool frequency WG sig improved stool form	This study demonstrates a differential impact of prebiotic action for WB and WG cereal, with a sig higher increase in bacterial no's effect for WG within the measured time frame
Vitaglioni et al. (12)	Italy 68 adults overweight/obese Av Age 38.5 y Av BMI 30	Pregnancy/lactation, medication (3 m), chronic illness, high fiber diet, probiotics, vitamins/minerals supplements, or complementary, and alternative medicines; fruit and vegetables >3 servings/d, >500 min exercise/wk	Whole grain wheat (Shredded Wheat) vs. refined wheat	Randomized parallel	16S rRNA gene sequencing	8 weeks	① 70 g/d (3 biscuits/d −8 g fiber) ② 70 g refined wheat crackers and toast (2.2 g fiber)	① Sig↑ Bacteroidetes and Firmicutes & sig ↓ Clostridium	① Sig 4-fold ↑serum dihydroferulic acid (DHFA) and 2-fold ↑ fecal ferulic acid (FA) with WG	WG wheat consumption significantly ↑ excreted FA and circulating DHFA. Bacterial communities influenced fecal FA & were modified by WG wheat consumption.
Freeland et al. (13)	Canada 40 adults—pre-diabetic (↑ insulin) Av age 29 y Av BMI 26	Antibiotics (3 m)GI, diabetes, hyperlipidaemia or a high-fiber diet.	Wheat bran fiber	Randomized parallel	n/a	1 year	① 60 g All Bran Original (24 g fiber) ② 49 g Rice Krispies (0.5 g fiber)	Not measured	① Sig ↑ plasma butyrate, acetate & GLP-1 in participants between 9 and 12 months.	Sustained ↑ in wheat fiber intake ↑ plasma butyrate & GLP-1 conc^n^ in hyperinsulinaemic participants, but it takes 9–12 months for these changes to occur.
Neacsu et al. (14)	UK 8 healthy adults Age 18–55 y BMI 18–30	Prescribed medication, use of nutritional supplements, smoking, antibiotics (3 M)	Wheat Bran breakfast cereal	Non-randomized acute	n/a	Single test meal 2 week washout	① 40 g All Bran Original (11 g fiber) ② 120 g All Bran original (33 g fiber)	Not measured	Sig ↑ plasma, urine and fecal SCFA & butyrate from ①&② Sig ↑ plasma ferulic acid by 5 h No sig differences between treatments	Significant increase in fecal butyrate after consumption of a 40 g bowl All Bran suggest that regular consumption of a wheat bran breakfast cereal will help support a healthy gut environment.
Deroover et al. (15)	Belgium 10 adults Age 18–65 y BMI 18–27	Pregnancy/lactation, GI disease, Anemia, Antibiotics, prebiotics, and probiotics (1 M)	Wheat bran effect	Randomized crossover	n/a	1 day each Washout 4 weeks	10 g labeled inulin plus ① 20 g wheat bran ② 20 g wheat bran ↓ particle size ③ 20 g pericarp enriched wheat bran	Not measured	Labeled fermentation markers appeared in breath & plasma around 3 h 45 min after consumption & continued for 8 h. No effect of bran particle size	Fermentation of a readily fermentable substrate increased plasma SCFA for about 8 h, suggesting that a sustained increase in plasma SCFA concentrations can be achieved when a moderate dose of fermentable carbohydrate is administered 3x per day
McIntosh et al. (16)	Australia 28 adult males Age 40–65y	Regular use of drug therapy, medication, or supplements that may interfere with bowel function, major illness	High fiber intake from wholegrain wheat vs. wholegrain rye—bread, crispbreads, and breakfast cereals	Randomized crossover	n/a	4 weeks each Washout—length not stated	① 21 g wheat fiber from cereal foods ② 21 g rye fiber from cereal foods ③ 6 g fiber as refined cereal foods	Not measured	Sig ↑ fecal weight with ① & ② & small sig ↓ fecal pH. Sig ↑ Butyrate with ② Sig ↑ propionate with ① Sig ↓ insulin & glucose response to breakfast meal with both ① & ②	Both high-fiber rye and wheat foods were equally effective in improving measures of bowel health. Rye foods ② more effective at ↑ plasma enterolactone and fecal butyrate
**BARLEY**										
Martinez et al. (17)	USA 28 adults (11 M and 17 F)	Antibiotics (3 M) GI disorder, antihypertensives, lipid lowering or other regular drug therapy	Barley vs. brown rice or combination of the two	Randomized cross over	16 S rRNA gene sequencing Joint Genome Institute database used to identify large-bowel associated bacteria with b-glucanase encoding activity.	4 weeks eachWashout 2 weeks	① 60 g whole grain barley flakes (18.7 g fiber) ② 60 g brown rice flakes (11.5 g fiber) ③ 60 g equal mix of the two (30 g fiber)	All ↑ bacterial diversity with Sig ↑ Firmicutes (Roseburia, Dialister, Eubacterium) & actinobacteria (Bifidobacteria) & Sig ↓ Bacteroidetes	No effect on SCFA Substantial individual variation in response Sig improvement in inflammatory responses & glycaemia with WGB & BR combined	Short-term intake of whole grains induced compositional alterations of the gut microbiota that coincided with improvements in physiological measures related to metabolic dysfunctions in humans
Nilsson et al. (4)	Sweden 20 adults (10 M and 10 F) Age 19–30 y Av BMI 22.1	Not reported	Barley vs. refined wheat	Randomized crossover Washout 1 week	n/a	1 evening test meal followed by standard white wheat bread breakfast	① WWB + Barley Dietary Fiber (BDF) (9.8 g fiber) ② Spaghetti + BDF (9.8 g fiber) ③ Spaghetti + BDF (19.6 g fiber) Spaghetti + oat DF (9.8 g fiber) Barley porridge (8.5 g fiber)	Not measured	Sig higher breath H_2_ after ②,③ & ③ with double BDF produced sig higher breath H_2_ cp to or ② Plasma SCFA sig higher after compared to barley porridge	Plasm propionate & butyrate -ve related to glucose response suggesting that SCFA derived from colonic fermentation are likely to be involved in modulating glucose response.
Nilsson et al. (18)	Sweden 15 adults (10 M and 5 F) Av age 25.6 y Av BMI 22.5	Antibiotic or probiotic use (2 W)	Barley vs. white bread	Randomized crossover Washout 1 week	n/a	8 individual meals	8 evening test meals with kernel based barley breads providing varying amounts of dietary fiber (13.7–30 g fiber)	Not measured	Sig ↑ plasma butyrate -measured following morning with High amylose barley and high ß-glucan barley Sig reduction in blood glucose response to test breakfast between 28 and 36%	The results of this study show that it is possible to increase the colonic production of SCFA in a semi-acute perspective (i.e., from an evening meal to the following morning) by choice of cereal foods rich in barley DF and RS.
Nilsson et al. (19)	Sweden 20 adults (3 M and 17 F) Av age 64 y Av BMI 23.6	Non-smoker, no metabolic disorder or illness Antibiotics (2 w) or probiotics (2 w)	Barley bread vs. white wheat bread	Randomized crossover	n/a	3 daysWashout 2 weeks	① 233 g white wheat bread (9.7 g fiber) ② 338 g barley bread (37.8 g fiber)	Not measured	② sig ↑ gut hormones, sig ↓ glucose & insulin response to test breakfast ② sig ↑ metabolic markers of microbiota activity—breath hydrogen, plasma SCFA, and acetate	Intake of barley bread for 3 d markedly increased gut fermentation activity suggesting that the metabolic benefits are related to gut fermentation of the DF fraction in Barley bread
**RYE**										
Graston et al. (20)	Finland 17 adults (8 M and 9 F) Av age 42 Av BMI 25.3	Not reported	Rye bread vs. white wheat bread providing 20% energy intakes	Randomized crossover	n/a	4 weeks Washout 4 weeks	① Rye bread (5.5–6.5 servings, 17.4–22.2 g fiber) ② white wheat bread (4.5–5.5 servings, 3.9–5.1 g fiber)	Not measured	Sig ↑ fecal weight & sig ↓ transit time on Rye bread No sig diff in total fecal SCFA Butyrate in feces higher in men on Rye bread phase	Consumption of rye bread in normal amounts improves bowel function. Effects on bacterial activity need evaluation in larger study population
Vuholm et al. (21)	Denmark 70 adults (32 M and 38 F) Av age 51 Av BMI 27.8	Smoking, GI disorders, diabetes or CVD, pregnancy or lactation, antibiotics (3 M) pre- or probiotics (1 M)	Rye wholegrains or wheat wholegrains vs. refined grain control	Randomized parallel	16S rRNA gene sequencing using The Greengenes database for reference	6 weeks	① Rye (124 g whole grains) ② Wheat (145 g whole grains) ③ Refined grains (5g wholegrains)	No effect	Fecal Butyrtae sig ↑ with both ① & ②	Regular consumption whole grain rye or wholegrain wheat affected fecal butyrate & GI symptoms in overweight adults and can be included in the diet equally to maintain gut health
Lee et al. (22)	Sweden 21 adults Av. age 38.6 y Av BMI 24.9	Diabetes, hyperlipidaemia, thyroid or metabolic disease, eating disorders, pregnancy, lactation, allergies, smoking	Wholegrain rye porridge + inulin/wheat gluten	Randomized crossover	n/a	Single test meal	① 40 g rye (7.1 g fiber) ② 55 g rye (9.7 g fiber) ③ 40 g rye + 9.3 g inulin/gluten (15.5 g fiber) 40 g rye + 6.6 g inulin/gluten (12.6 g fiber) 40 g rye + 3.9 g inulin/gluten (10.4 g fiber)	Not measured	Sig higher breath H_2_ with compared to wheat bread or	Whole grain rye suppressed hunger compared to wheat bread but there were no additional effect from adding inulin or gluten. Large dose dependent ↑ breath H_2_ in response to fiber
**RICE**										
Nemoto et al. (23)	Japan 36 adults (14 M, 22 F) Age 22–67 years	Food allergy, serious illness, antibiotics or agent known to influence bowel condition	FBRA-Fermented Brown Rice and rice bran	Randomized crossover	16S rRNA gene sequencing	2 weeks each Washout 12 weeks	① 21 g FBRA (5 g fiber)−7 g after each meal ② 21 g control (0.4 g fiber)	Change to total bacterial no's not sig diff between group ① & ② or after any test period. SCFA production also failed to show any sig differences.	No significant effects. *In vitro* testing from 6 participants showed ↑ bifidobacteria & ↑ total SCFA, lactate & acetate with FBRA	FBRA increased production of SCFA and bifidobacteria *in vitro*, however it remains unclear as to whether this has prebiotic effects in the intestinal environment.
Sheflin et al. (24)	USA 7 adults Av age 42 y BMI 22–29	No history food allergy, no cholestertol lowering medication of NSAID's, pregnancy or lactation, smoking, antibiotics (3 M), probiotics (3 M)	30 g heat stabilized rice bran	Randomized parallel	16S rRNA gene sequencing	28 days	① 1 study meal & 1 study snack daily (30 g rice bran)	Sig ↑ bifidobacteria, ruminococcus species and 6 others	Sig ↑ branched chain fatty acids & butyrate	This pilot study supports that consumption of 30 g rice bran can positively affect the gut microbiota & its metabolites
**OAT**										
Connolly et al. (25)	UK 30 adults (11 M, 19 F) mild hyperglycaemia or mild hypercholesterolaemia Av age. 42 y Av BMI 26.4	Pregnancy/lactation, food allergy, antibiotics (6 w), chronic illness, lipid lowering drugs, GI disorder, Drugs affecting GI, substance misuse, alcoholism	Whole grain Oat (WGO) Granola	Randomized crossover	16S rRNA gene sequencing & FISH	6 weeks each Washout 4 weeks	① 45 g of WGO granola (2.8 g fiber, 1.3 g ß-glucan) ② 45 g non-whole grain (NWG) breakfast cereal (flaked corn, 1.3 g fiber/serve)	① Sig ↑ bifidobacterial, lactobacilli & total bacterial count ② Sig ↓ bifidobacteria & total bacterial count	No Sig effect fecal SCFA ① Sig ↓ total chol, near sig ↓ fasting glucose ② Sig ↑ total & LDL chol	Dietary WGO ingestion had an appreciable Impact on the composition of the human gut microbiota, and significantly reduces plasma TC & LDL-C
Valeur et al. (26)	Norway 10 adults 22–49 y Av BMI 23	Pregnancy, Chronic illness Gi disease Antibiotics (1 M)	Oatmeal porridge	Non-randomized single arm	n/a	8 days	60 g oatmeal (8·5 g fiber, including 4·7 g β-glucans)	Not measured	No sig effect of fecal SCFA Sig ↓ fecal levels of β-galactosidase & urease suggesting impact on microbial activity	Ingestion of oatmeal porridge daily for 1 week ↑ some metabolic markers of increased microbiota activity however colonic fermentation capacity & fecal SCFA were unaltered.
**MAIZE**										
Carvalho-Wells, (27)	UK 32 adults (11 M, 21 F) Av. age 32 y Av BMI 23.3	Pregnancy/lactation, antibiotics (6 m), GI drugs or laxatives, anemia, hyperlipidaemia	Whole grain maize cereal	Randomized crossover	16S rRNA gene sequencing	3 weeks each Washout 3 weeks	① 48 g WG maize breakfast cereal (14.2 g fiber) ② 48 g maize cereal (0.8 g fiber)	① Sig ↑ bifidobacteria and non-sig ↑ lactobacilli & Atopobium levels. ② Non-sig changes to bifidobacteria, lactobacilli, and Atopobium levels	Treatment effects not sustained following wash out period. No sig changes to fecal SCFA, bowel habit data, fasted lipids/glucose, and anthropometric measures	Present study showed a prebiotic effect from a WG maize cereal, which resulted in a beneficial shift in the fecal microbiota
**MIXED WHOLE GRAIN DIETS (PREDOMINATELY WHEAT)**										
Walker et al. (28)	UK 14 obese males Av age 54 y Av BMI 39.4	No GI disease Antibiotics (6 M) No weight loss (4 M)	Resistant Starch vs. NSP (wheat bran) or High protein weight loss diet	Randomized crossover	16S rRNA gene sequencing and qPCR	3 weeksWashout not stated	① Maintenance (27.7g NSP, 5g RS) ②RS III (25.5g RS, 16g NSP) ③NSP (41.9g NSP, 2g RS) HP WL (25g NSP, 2g RS)	② Sig ↑ ruminococcus & eubacterium ③ No major change in fecal microbiota sig ↓ eubacterium & collinsella	Fermentation metabolites not measured. RS almost totally digested (96%) in 12 of 14 participants Soluble NSP 90% digested Insoluble NSP 66% digested	Increased intake of RS gave substantial increases in species in colonic microbiota. However the lack of change resulting from NSP may be due to smaller increase in NSP intake (1.5x) compared to a 4.8-fold increase in RS intake on test diets.
Salonen et al. (29)	UK 14 males metabolic syndrome Av age 53 y Av BMI 39.4	GI disease Antibiotics (6 m)	Resistant starch vs. wheat bran	Randomized crossover	16S rRNA gene sequencing & qPCR	3 weeks eachWashout 1 week	① Control diet 27.7 g fiber ② High RS ③ High NSP (wheat bran) Low carb weight control	Diversity of microbiota was sig lower ① & ② but ↑ sig with ③ (wheat bran) Firmicutes fell ~3-fold on Changes to bacterial abundance were Smaller increase in abundance on NSP diet but may be due to smaller ↑ in fiber intake cp to control diet (1.5x cp to 4.8 x on RS)	Fecal acetate, Propionate & butyrate ↑ on ③ Chemical analysis of fecal samples showed soluble NSP digestibility to be around 88–90% Insoluble NSP digestibility = 66%	NSP and RS, affect distinct bacteria, and have different impact on the community ecology of the human gut. RS reduced diversity while increasing specific bacterial types while wheat bran had a more modest impact on bacterial abundance while increasing diversity of the microbiota.
Christensen et al. (30)	Denmark 79 overweight/obese post-menopausal women Age 45–70 y BMI 27–37	Not reported	Whole grain vs. refined grain on energy restricted diet	Randomized parallel	16 S rRNA gene sequencing	12 weeks 2 week run in	① 105 g of whole grain products ② 105 g refined grain products	Sig ↑ Bifidobacterium with ① whole wheat groupSig ↓ Bacteroides with ② RW group	Fermentation metabolites not measured. Sig -ve correlation between bacteroides abundance & % fat mass & trunk fat Sig +ve correlation between Bifidobacterium & % fat mass & trunk fat	This study, consistent with other studies, supports the prebiotic potential of whole wheat grain products.
Vetrani et al. (5)	Italy 54 adults overweight/obese (23 M, 31 F) Av age 58 y Av BMI 32	Diabetes, renal failure, liver abnormalities, anemia, chronic disease, alcohol abuse	Wholegrain products, e.g., bread, breakfast cereals, pasta etc. Mainly wheat some rye	Randomized parallel	n/a	12 weeks	① Wholegrain (40g total fiber, 29g cereal fiber) ② refined grain (22g total fiber, 12 g cereal fiber)	Not measured	Sig ↑ plasma propionate with ① which was +vely & sig associated with cereal fiber intake	Habitual consumption wholegrain foods may promote colonic fermentation of fiber, and increased propionate levels may help to modulate insulin response.
Cooper et al. (31)	USA 46 adults (21 M and 25 F) Av age 23.4 y Av BMI 23.4	Diabetes, GI disease/IBS, laxatives, antibiotics (3 M) smoking, pregnancy, lactation	Wholegrain products e.g., bread, breakfast cereals, pasta etc. Mainly wheat some corn & rice	Randomized parallel	16S rRNA gene sequencing	6 weeks	① 6 servings wholegrains (13.7g fiber) ② 6 servings refined grains (4.2g fiber)	No sig changes but trends toward ↑Akkermansia & lactobacillus& Erysipelotrichales *N.B. Fecal samples analyzed from only 28 participants*	Not measured	Microbial analysis lacked power due to small sample size and requires further research
Ampatzoglou et al. (32)	UK 33 adults (12 M, 21 F)	Chronic illness/medication Substance/alcohol misuse Antibiotics(3 m) Probiotics (3 m) Habitual high fiber/wholegrain	Commercially available whole grain pasta, rice, snacks, and breakfast cereals	Randomized crossover	FISH	6 weeks Washout 4 weeks	① High whole grain (>80 g/d) ② low whole grain (<16 g/d)	No sig effect	Fermentation metabolites not measured. No sig effects Trends toward ↓ BMI, blood glucose, & ↑ fecal weight with ①	Little effect of WG consumption on blood biochemical markers, body composition, BP, fecal measurements, or gut microbiology. This may be due to impact commercial production processes on levels of undigestible fermentable carbohydrates
Ross et al. (33)	Switzerland 17 adults (6 M and 11 F) Age 20–50 y BMI 19–28	Healthy, no medication, normal blood lipids, no recent antibiotics, non-smilers	Commercially available wholegrain foods inc. wheat, oats, and brown rice	Randomized crossover	Quantitative PCR	2 weeks Washout 5–7 weeks	①150 g wholegrain foods (34 g fiber) ② 150 g refined grain foods (19 g fiber) Both 2/3 wheat + oats & rice	① sig ↑ clostridium leptum Trend toward ↑enterococcus	Fermentation metabolites not measured. ① sig ↑ stool frequency & trend toward ↓ LDL cholesterol 2 weeks too short to see full effects on gut microbiota.	Small changes in fecal microbiota after 2 weeks suggest that longer term wholegrain diets could have greater effects on gut microbiota. This requires further study in longer trials with greater no's participants
Tap et al. (34)	France 19 adults (9 M and 10 F) Age 18–30 y BMI 18.5–25	Antibiotics (3 M) Laxatives (3 M) No history GI problems or taken GI medications (3 M)	High vs. low fiber intake mixed diet	Randomized crossover	16S rRNA gene sequencing & qPCR	5 days Washout 2 weeks	① 40g fiber ② 10g fiber	① Sig ↓ Escheria coliLow level of microbial richness at outset was associated with sig microbiota change with ①	Fermentation metabolites not measured.	Short-term change in dietary fiber impacts gut microbiota differently within participants—sig change seen in all individuals.
Vanegas et al. (35)	USA 81 adults (49 M and 32 F postmenopausal) Age 40–65 y BMI <35	Supplement use, weight loss diet, NB. probiotics/supplements stopped 30 days prior to trial Alcohol abuse Antibiotics (3 M) Medication use	Whole grains vs. refined grains Wheat main source WG	Randomized parallel	16S rRNA gene sequencing—Greengenes reference database & USEARCH program	6 weeks	①WG diet 40 g fiber/day (16 g fiber/1,000 kcal) ②RG diet 21 g fiber/day (8 g fiber/1,000 kcal)	① sig ↑ Lachnospira & ↓ Enterobacteriaceae	① ↑ bowel movement freq & stool weight ① Sig ↑ in stool total SCFA's & acetate	Short-term consumption of wholegrains improves bowel function and has modest positive effects on gut microbiota, SCFA and innate immune response. Prolonged intervention may give more pronounced changes in microbiota &inflammatory markers.
**IMPACT OF REDUCING CARBOHYDRATE/FIBER INTAKE**										
Lappi et al. (36)	Finland 51 adults (25 M and 26 F) with metabolic syndrome Av Age 60 y Av BMI 31 + at least 3 other features metabolic syndrome	BMI >40 Very high blood lipids Diabetes, liver, thyroid, renal disease Alcohol abuse IBS.	Replacement rye bread with refined wheat bread Same total grain intake but different quality	Randomized parallel	16S rRNA gene sequencing and qPCR analysis	12 weeks	① High fiber Rye bread (24 g fiber) ② refined wheat bread (19 g fiber)	② Sig 16% ↑ Bryantella Formatexigens ② 37% ↓ in Bacteroidetes—due to removal of rye breads A substantial individuality characterized microbiota responses—most unidirectional but some not responders	Fermentation metabolites not measured.	Intentional modulation of the gut microbiota by withdrawal or supplementation is not straightforward due to individual variations in microbiota. Changing from high to low wholegrain diet did not produces difference sin gut microbiota in individuals with metabolic syndrome
Duncan et al. (37)	UK 19 obese but healthy adults Av. Age 36.7y Av BMI 35.4	No history of gastrointestinal problems. No antibiotics or drugs known to influence microbiota	Reduction in carbohydrate and fiber intake	Randomized crossover	FISH and 16S sRNA gene sequencing	4 weeks Washout 3 days	① High protein/medium CHO (164 g CHO & 11.7 g NSP/day) (HPMC) ②High protein/low CHO (24 g CHO & 6.1 g NSP/day) (HPLC) ③ Maintenance (M) (399 g CHO & 27.9 g NSP/day)	Sig change in bacterial no's Roseburia >> Eubacterium rectale >> Bifidobacteria >>	Sig ↓ in fecal total SCFA (50%) & butyrate (75%) with decreasing CHO & fiber >> Total fecal SCFA correlated positively with fiber intake	Butyrate production Is largely determined by fermentable carbohydrate in the diet. Long term consequences of low SCFA in colon are unknown however consideration to adequate supply of fermen table substrates should be given if low carb diet to be followed for long periods
Brinkworth et al. (38)	Australia 91 overweight or obese adults Age 24–64 years BMI 26–44	Liver, cardiovascular, peripheral vascular, respiratory, GI, renal or hepatic disease or a malignancy. Regular use drug therapy, medication or supplements such as laxatives or antibiotics	Comparison high or low carb energy restricted diets	Randomized parallel	Selective plating for bifidobacteria, lactobacilli, total anaerobes, and E. coli, coliforms and total aerobes & visual counting	8 weeks	① High carb (46% CHO, 32 g fiber) ② Low carb (4% CHO, 13 g fiber)	Sig ↓ fecal bifidobacteria in low CHO group	Fecal acetate & butyrate sig ↓ (30–60% lower) on the LC diet	Short term consumption of a low carb diet had a negative impact on bowel health: including lower stool mass, less frequent bowel movements, reduced large-bowel fermentation (↓conc^n^) & excretion of fecal SCFA inc butyrate, & unfavorable shift in fecal microflora composition (↓ bifidobacteria)
**WHEAT BRAN EXTRACT—ARABINO-XYLAN-OLIGOSACCHARIDE (AXOS)**										
Maki et al. (39)	USA 55 adults Age 18–75 y BMI 18.5–35.0	Lipid lowering medication	Wheat bran extract AXOS	Randomized crossover	FISH	3 weeks Washout 2 weeks	AXOS at 0 (control), 2.2 g, or 4.8 g/d as part of wheat based ready-to-eat cereal −2 × 44 g servings cereal daily	Sig ↑ bididobacteria with 4.8 g AXOS provided as 2 × 2.4 g doses in a wheat based breakfast cereal Bifidobacteria levels ↑ in a dose-dependent manner 4.8 >2.2 g>control	Sig ↑ in plasma ferulic acid with 2.2 and 4.8 g AXOS—again dose dependent trend 4.8>2.2 g No change to Acetic acid or proprionic acid, butyric acid ↓ with increasing AXOS	Sig ↓ LDL cholesterol with 4.8 g/d
Johansson Boll et al. (40)	Sweden 19 adults (9 M and 10 F) Av age 23 Av BMI 22	Smoking Antibiotics (2 w) Probiotics (2 w) Food allergise Metabolic disorders	Wheat Bran extract AXOS	Randomized crossover	n/a	Single test meal Washout 1 week	①White wheat bread (WWB) (1.2 g RS) ②WWB + AXOS (8.9 g) + RS (6.6 g) ③WWB + hiAXOS (18.9 g), RS (1 g) WWB + RS (15 g RS)	Not measured	Sig dose dependent ↑ breath Hydrogen with AXOS ② & ③ Sig dose dependent ↑ SCFA & Butyrate with ② & ③ No sig diff in post prandial glucose or insulin after breakfast meal but improved insulin sensitivity index with AXOS	An AXOS rich substance has the potential to influence overnight glycaemic regulation and gut fermentation in healthy young adults.
Windey et al. (41)	Belgium 29 adults Age 19–44y BMI 18.7–24.3	Abdominal surgery, Liver or kidney failure, GI conditions. Pregnancy, lactation, Drugs affecting GI tract (14 days), Antibiotics (1 M)	Wheat bran extract (75% AXOS)	Randomized crossover	DGGE, plus real-time PCR GenBank DNA database	3 weeks Washout 3 weeks	①WBE 10 g/day (2 × 5 g sachet) AXOS = 7.5 g avDP5 ② 10 g (2 × 5 g sachet) maltodextrin (placebo)	Sig ↑ bifidobacteria with ① WBE No diff in fecal SCFA	Sig ↓ colonic fermentation protein with ① WBE	Supplementing the diet with WBE clearly altered fermentation in the colon & selectively stimulated growth of bifidobacteria.
Cloetens et al. (42)	Belgium 12 adults (6 M and 6 F) Av age 24 Av BMI 21.9	No GI disease Antibiotics (3 M)Medication affecting GI tract (3 M)	AXOS avDP 15 in varied doses + 3 stable isotopes to measure gastric emptying, transit time & colonic NH_3_ metabolism	Randomized crossover	n/a	Single test meal Washout 1 week	①AXOS 0.2g, ② AXOS 0.7g ③AXOS 2.2g AXOS 4.9g control Given in single daily test meal	Not measured	Gut motility not affected. Both ③ & (2.2 g and 4.9 g AXOS) resulted in: Sig ↑ breath hydrogen & sig ↓ urinary nitrogen Tendency to ↑ fecal nitrogen	A minimal does of 2.2 g AXOS favorably modulates colonic bacterial metabolism with increases in indicators of fermentation and bacterial growth.
Cloetens et al. (43)	Belgium 22 adults	Not reported	AXOS—same dose 2.25 g but different degree polymerisation	Randomized parallel	RT PCR—bifidobacteria, lactobacteria, and eubacteria	2 weeks	①AXOS 2.25 g av DP 9 ② AXOS 2.25 g avDP 15	Sig ↑ bifidobacteria with ① 2.25 g AXOS avDP9Trend to ↑ Bifidibacteria with ② but not significant change from baseline	Not measured	Bifidogenic properties of AXOS are affected by the degree of polymerisation with shorter molecules being more bifidogenic.
Cloetens et al. (44)	Belgium 20 adults (6 M and 14 F) Av age 24 Av BMI 20.9	GI complaints, antibiotics (3 M), drugs influencing GI transit (3 M), Abdominal surgery, pregnancy	Wheat bran extract (AXOS av DP 6) in orange juice	Randomized crossover	RT PCR—Bifidobacterium, Bifidobacterium adolescentis, total bacteria, Lactobacillus, Roseburia–Eubacterium rectale, and enterobacteria	3 weeks Washout 4 weeks	① AXOS 10g (2 × 7 g sachet) ② 10 g maltodextrin (2 × 7 g sachet)	Sig ↑ bifidobacteria at 2 & 3 weeks of AXOS intake Bifidobacteria also ↑on placebo but AXOS effect was sig > than placebo effect Increase most pronounced in participants with lowest starting levels of bifidobacteria	Fecal SCFA not measured. No influence of plasma folate, Vit A or minerals. No sig diff blood lipids.	This higher dose of AXOS was well tolerated and showed significant prebiotic activity.
Francois et al. (45)	Belgium 63 adults (33 M and 30 F) Av age 42 y Av BMI 23.3	Low energy/extreme diet (6 W), Antibiotics (3 M), drugs or supplements affecting GI tract (2 W), abdominal surgery/GI disease, excess alcohol/smoking, pregnancy, lactation	Wheat bran extract (79% AXOS, Av DP 5) in soft drink	Randomized crossover	FISH	3 weeks Washout 2 weeks	① 0 g WBE ② 3 g WBE(2.4 g AXOS) ③ 10 g WBE (8 g AXOS) (split over 2 doses)	③ Sig (2-fold)↑ bifidobacteria ② trend to ↑bifidobacteria (1.3-fold) but not sig. (p0·065)	③ ↑ fecal total SCFA by 8% (acetic, propionic & butyrate) ② sig ↑fecal propionic acid level	An intake of 10 g WBE/day (8 g AXOS) increased production of SCFA, reduced protein fermentation & increased fecal bifidobacteria levels, and is well tolerated.
Scarpellini et al. (46)	Belgium 13 adults (6 M and 7 F) Av Age 32.2 y Av BMI 23	GI disease Antibiotics (3 M) Pregnancy	Wheat bran extract (AXOS) in drink	Randomized crossover	n/a	48 h Washout 1 week	① AXOS 4x 9.4 g/day ② maltodextrin 4 × 10 g	Not measured	Sig ↑ breath hydrogen indicating ↑colonic fermentation on day 1&2 with AXOS	Acute AXOS administration is associated with increased colonic fermentation
Hamer et al. (47)	Belgium 20 adults (6 M, 13 F) Av age 23 y Av BMI 23	Not reported	AXOS 10 g/day	Non-randomized intervention	n/a	3 weeks	AXOS 10 g/day for 3 weeks	Not measured	179 different VOC's identified in fecal samples. With 24 present in >70% samples. Shift away from protein fermentation seen.	AXOS has considerable impact on colonic fermentation mainly by suppression of proteolytic activity.
**OTHER ISOLATED FIBERS**										
Abell et al. (48)	Australia 46 adults (16 M, 30 F) Age 25–66 y Av BMI 26	Antibiotics (3 m) Smokers GI disease	NSP (bran fiber) vs. NSP + Resistant starch (barley fiber and HiMaize) given as dietary supplements	Randomized crossover	DGGE of 16S rRNA gene fragments	4 weeks each Washout 2 weeks	①Normal diet ②NSP (25 g fiber & 1 g RS) ③NSP + RS (25 g fiber + 22 g RS)	Sig shift in bacterial DNA in fecal samples on both NSP and NSP+RS diets ↑in Ruminococci species with NSP+RS	Sig ↑ fecal total SCFA acetate, propionate, and butyrate NSP+RS > NSP	This study suggests R. bromii-related organisms to be an important player in starch colonization and digestion. Longer-term dietary intervention on the colonic microbiota is needed to elicit a stable shift in microflora

*M, male; F, female; BMI, body mass index; GI, gastrointestinal; WG, wholegrain; WB -wheat bran; NSP, non-starch polysaccharide; AXOS, arabinoxylooligosaccharides; WBE, wheat bran extract; SCFA; short chain fatty acids; CHO, carbohydrate; RS, resistant starch; DP, degree of polymerization; VOC, volatile organic compound; FISH, fluorescence in situ hybridization; DGGE, denaturing gradient gel electrophoresis; PCR, polymerase chain reaction, real time (RT), or quantitative (qPCR)*.

### Data Extraction

For the studies selected for inclusion, general study characteristics (author, year of publication, study design, and length of study), characteristics of study population (age, gender, BMI), experimental manipulation (fiber type, amount, and length of manipulation), study outcomes (e.g., change to bacterial abundance or species diversity, and fermentation metabolites), and any other outcomes such as fecal weight, frequency, change to lipid levels etc. were extracted. A summary table of the study characteristics and findings is shown in Table 2. The studies were categorized and discussed according to the fiber manipulation, such as whole diet wholegrain studies, wheat bran, oat bran, or specific fiber sub-fractions such as AXOS or arabinoxylans.

## Results

### Summary of Studies and Their Characteristics

The flow of included studies is outlined in Figure 1. Details of the studies, and their characteristics is presented in Table 2. A summary of the extracted outcomes is reported in Table 3 in relation to fiber type and direction of change.

**Table 3:** Summary of findings for the short-term effect of increasing cereal fiber on gut microbiota outcomes in health adults.

**Effect on microbiota**	**Total studies**	**Significant changes bacterial abundance**	**Significant changes to fermentation metabolites**	**No effects**	**Notes**
**SHORT-TERM EFFECTS OF FIBER MANIPULATION**					
Whole diet mixed grain fiber (predominantly wheat)	10	6 sig 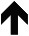 abundance and/or diversity	5 sig 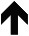 SCFA and/or metabolic markers of fermentation	2 no sig effects bacterial no's−1 due to small sample size	2 studies did not measure bacterial change 5 studies did not measure fermentation metabolites
Reduction of carbohydrate and/or fiber in diet	3	3	2	–	1 study did not measure fermentation metabolites
Wheat fiber/bran	8	3 sig 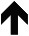 bacterial types and/or diversity	6 sig 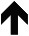	1 no effect on bacterial abundance 1 no change to SCFA	4 studies did not measure change to bacterial abundance
Barley fiber	4	1 sig 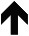	3 sig 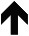	1 no effect on SCFA despite ↑ bacterial no's	3 studies did not measure bacterial change
Oat fiber	2	1 sig 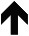 with fiber and sig 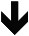 refined grain	1 change to metabolic markers of fermentation	2 no sig effect on fecal SCFA	1 did not measure bacterial change
Maize fiber	1	1 sig 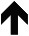	Not measured	1 no effect fecal SCFA	Sig 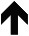 bifidobacteria
Rye fiber	5	1 shifts in bacterial phylotype	2 sig 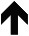 butyrate and breath H_2_1 sig 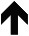 butyrate in men only	2 showed no effect on total microbiota abundance	3 studies did not measure change to bacterial abundance 1 study did not measure fermentation metabolites
Rice fiber	3	2 sig 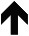	1 sig 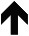 SCFA	1 no change to bacterial abundance 2 no effect on fecal SCFA	
Wheat AXOS	9	5 sig 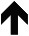 target species	6 sig 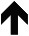 breath H_2_, plasma ferulic acid or SCFA	1 no effect fecal SCFA—poss due rapid fermentation and absorption	4 studies did not measure change to bacterial abundance 2 studies did not measure fermentation metabolites

The 40 included studies included a total of 1,308 participants. Of these studies, 26 utilized a randomized crossover design, 11 were parallel randomized trials, and 3 were non-randomized interventions. Seven studies involved feeding single test meals in a laboratory setting, and the remainder involved foods consumed at home. Intervention length ranged from 1 meal to 1 year.

A total of 4 studies compared the effects of more than one fiber type, with the remaining 36 studies examining a single fiber source compared with low fiber foods or habitual dietary intake. Wheat was the most commonly studied fiber with 9 studies on wholegrain intake (predominantly wheat but some other grain fiber), 8 exploring wheat fiber or bran exclusively, and a further 9 utilizing wheat bran AXOS. Five studies reported on rye fiber. While the prebiotic effect of isolated beta-glucans on the gut microbiota has been studied extensively, only 2 studies were identified examining the effects of intact oat fiber on the gut microbiota. Barley, rice and maize accounted for the remaining 8 studies.

### Impact of Wheat Fiber or Wheat Bran on Gut Microbiota

Wheat fiber or bran is the hard outer layers of the wheat kernel. Wheat bran is particularly rich in dietary fiber and essential fatty acids, and also contains appreciable quantities of starch, protein, vitamins, and dietary minerals. Wheat is widely consumed and a significant contributor to fiber intakes in Western Societies, with approved health claims for digestive health in many countries including the European Union (EFSA 2010 j.efsa.2010.1817), Canada (Health Canada), USA (US FDA Laxative Monograph), and Australia (Food Standards Australia New Zealand 2014). It is also the most studied in relation to its impact on the gut microbiota.

In total, 8 studies examined the impact of manipulating wheat on the gut microbiota, of which 5 increased fiber at a breakfast meal (11–15). Three examined the effect of whole day diet interventions (16, 28, 29), of which two conducted different types of analysis on fecal samples from the same study sample and intervention (28, 29). One analysis identified change in key dominant bacteria phylotypes (28), while the other more specific analysis identified change to individual bacterial species (29). Wheat fiber provision ranged from 5.7 g to 21 g/day and wheat bran from 13 g to 28 g/day. Both bacterial abundance and fermentation metabolites were measured in 4 studies, and the remaining 4 studies measured only change to the metabolites of bacterial fermentation. Six of the 8 studies (11–14, 29) showed significant effects on gut microbiota from wheat fiber or bran fiber consumption and 2 showed no effect (15, 28). Significant increases were reported both in terms of phyla: Bacteroidetes (12, 29); Firmicutes (12, 29); and Actinobacteria (29), and specific species: Bifidobacteria (11); Lactobacillus (11); Atopobium (11); Enterococci (11); Clostridia (11); Lachnospiraceae (29); Eggerthella (29); Collinsella (29); Corynebacterium (29); Bacteroides (29); and Prevotella (29).

Four interventions utilizing a single daily serving of wheat bran fiber at breakfast all demonstrated a significant prebiotic effect. Costabile et al. (11) examined the effect of consuming 48 g of wholegrain wheat cereal (5.7 g fiber) or a 48 g of wheat bran rich cereal (13 g fiber) daily for 3 weeks, and reported significant increases in Bifidobacteria following wholegrain consumption and significant increases in Lactobacilli and Enterococci after either cereal vs. baseline. Bacterial response following the wholegrain cereal was significantly greater compared to wheat bran, however the wholegrain cereal used had been specifically chosen compared to similar cereals for its ability to stimulate microbial growth. Change to bacterial abundance was enumerated using Fluorescence *in situ* Hybridization (FISH) which depends on the pre-selection of probes for specific bacterial types. Probes were selected to detect change to dominant members of the gut microbiota, and change to less dominant species reflecting wider benefits arising from fiber consumption may have not been captured. Both cereals significantly increased plasma ferulic acid levels with higher levels reported following consumption of wheat bran, and this was the first study to demonstrate in human participants that the regular consumption of wholegrain or wheat bran is followed by a slow and continuous release of phenolic acids into the bloodstream. Dietary fiber is rich in phenolic compounds, particularly ferulic acid, the majority of which is bound to the arabinoxylan present in the bran fraction of the wheat kernel and is released by microbiota action. The appearance of ferulic acid in plasma or feces can be used as a marker of bacterial fermentation in the colon, however levels will reflect all sources of dietary fiber consumed unless these are carefully controlled. Vitaglioni et al. (12) examined the effect of consuming 70 g of wholegrain wheat cereal (8 g fiber) compared to 60 g of refined wheat (2.2 g fiber) daily for 8 weeks and demonstrated significant increases in Bacteroidetes and Firmicutes, accompanied by a 4-fold increase in plasma ferulic acid and 2-fold increase in fecal ferulic acid among the wholegrain group compared to the refined wheat consumers. Wholegrain wheat was the unique source of ferulic acid in this study allowing differentiation between the two intervention groups.

Neacsu et al. (14) fed either a 40 g bowl of All Bran original cereal (11 g fiber) or a 120 g bowl of All Bran original (33 g) as a single test meal and then measured fermentation metabolites in plasma, urine, and feces over a 24 h period. Significant increases in total short chain fatty acids were measured in plasma, urine and fecal samples, following consumption of both 40 or 120 g wheat bran cereal, with no significant differences found between treatments. Additional unpublished data provided by the author, shows the largest increase to occur in fecal butyric acid, with a 2-fold increase over a 24 h period.

Freeland et al. (13) was the only study that ran for longer than 3 months. The intervention consisted of daily consumption of 60 g of wheat bran cereal (24 g fiber) for 1 year, compared to 49 g of low fiber cereal (2.2 g fiber). No other dietary restrictions were required. Metabolic profiling was undertaken at baseline and 3-monthly intervals for the duration of the intervention. Compliance was good and 20 g/day increase in fiber was achieved (38 g total fiber/day) compared to the control group (19 g total fiber/day). Fecal samples from this study were lost and so change to the microbiota was limited to plasma SCFA levels measured over an 8 h period following a test breakfast taken at baseline, 3, 6, 9, and 12 months post intervention. Plasma levels of butyrate were significantly higher for the wheat bran consumers at 9 months post-intervention vs. low fiber cereal consumers. There were no other significant effects at any other time points. The authors acknowledge the limitations of SCFA measurements in plasma, but conclude that sustained butyrate and Glucagon-like-peptide-1 levels (GLP-1—a peptide hormone which stimulates production of insulin thus lowering blood sugar) from 9 months onwards might provide a mechanism for the reduced levels of diabetes associated with high fiber intakes. Similar links between gut microbiota fermentation, SCFA, and modulation of blood glucose and insulin responses have also been made by acute trials (4, 18) lasting 12–14 h. The final breakfast intervention measured appearance of metabolites from labeled inulin (15) and whether the addition of wheat bran to inulin affected its appearance. The conclusion was that wheat bran had no additional benefit, however the study had a number of limitations in relation to wheat bran fermentation which are discussed later.

These studies demonstrate that a relatively low wheat fiber intake at a single time point (breakfast) can maintain a prebiotic effect, despite the presence of a mixed habitual diet. All 3 studies increasing wheat fiber over the whole day (16, 28, 29) showed substantial effects on species composition within the gut microbiota, and the results are presented in more detail in the whole diet section below (section Impact of Mixed Whole Grains on Gut Microbiota). Salonen et al. (29) compared effect of wheat bran vs. resistant starch and reported lower increases in individual bacteria with wheat bran consumption, but this was accompanied with a marked increase in overall microbial diversity.

### Impact of Barley Fiber on Gut Microbiota

Barley contains a mixture of both soluble (beta-glucans) and insoluble fibers giving it a diverse range of potential health benefits, including moderating blood cholesterol and provision of a food source for the gut microbiota. Similar to wheat, barley fiber also contains essential fatty acids, starch, protein, vitamins, and dietary minerals.

A total of 4 studies (4, 17–19) have investigated the impact of barley fiber on the human gut microbiota, 3 of which have been carried out by the same research group in Sweden over a period of 7 years (4, 18, 19). Barley was provided either as a single test evening meal (*n* = 2) or consumed freely as a barley bread or barley cereal, with fiber amounts varying from 9.8 to 19.6 g. All studies showed a substantial impact of barley fiber on markers of gut microbiota: either change to microbiota population (17) or fermentation metabolites (4, 18, 19). Only 1 of the studies (17) examined microbiota population and found significant increases in Firmicutes and Actinobacteria (specifically Roseburia, Dialister, Eubacterium, and Bifidobacterium) and a significant decrease in Bacteroidetes, The remaining 3 studies (4, 18, 19) measured markers of fermentation (breath hydrogen, plasma SCFA) and showed significant increases in total SCFA, butyrate and acetate, and significant increases in breath hydrogen following barley fiber consumption. All 4 studies demonstrated concomitant improvements in glycemic response attributed to the positive effects of fermentation metabolites.

### Impact of Rye Fiber on Gut Microbiota

Rye is a staple cereal across Central, Eastern, and Northern Europe, most often consumed as breads and crispbread, and providing a significant contribution to fiber intakes in these communities. Rye contains a mixture of soluble and insoluble fibers plus essential fatty acids, starch, protein, vitamins, and dietary minerals.

Of the 5 studies into the effect of rye intake on the gut microbiota, 4 were carried out in Scandinavia where rye breads and other products are a staple part of the everyday diet (20–22, 36), and the other carried out in Australia (16) compared the effects of both wheat and rye. Intake of rye fiber varied from 7.1 to 24 g daily. Only 2 studies measured the effects on microbiota abundance, both using 16S rRNA gene sequencing, and showing no effect on total bacterial abundance (21, 36). Lappi et al. (36) however reported significant shifts in bacterial composition with a significant (37%) decrease in Bacteroidetes on switching intake of wholegrains, predominantly rye bread, over to refined wheat products, with a parallel increase in Firmicutes (Clostridium sp.) and Actinobacteria (Collinsella and Atopobium sp.). Bacteria within the Bacteroidetes phyla are known to be able to utilize the arabinoxylan fiber fractions in rye (49), so a decline in Bacteroidetes when rye breads are removed from the diet is not surprising.

In contrast, 3 of the 5 studies (16, 21, 22) reported an increase in the metabolites of bacterial fermentation with significant increases in fecal butyrate (16, 21) or breath hydrogen (22). One study did not measure metabolites (36) and the other reported no change in fecal butyrate for women, but a significant increase in men (20). During this study the men consumed significantly larger quantities of the rye bread and as a consequence significantly more rye fiber (19.1 g fiber from the test bread compared to 13.5 g consumed by women). The authors determined that from this study it was not possible to conclude whether the differences in response to rye bread between women and men were due to different amounts of food consumed or to differences in fiber intake from breads and that further exploration with larger participant numbers is required.

### Impact of Rice Fiber on Gut Microbiota

Like other cereals, the rice grain is enclosed in an outer bran layer, rich in fiber minerals and vitamins and antioxidants. Rice bran contains a higher levels of oils compared to other cereal brans, and so is often removed from the grain to reduce risk of rancidity and improve storage longevity, as a result reducing the range of fiber rich rice products commonly available for consumption.

Only 3 studies were identified exploring the links between rice fiber and the gut microbiota. One was carried out in Japan (23), and the other 2 in the USA (17, 24), however it should be noted that one of these was a pilot trial involving just 7 participants (24). The Japanese study (23) used Fermented Brown Rice by Aspergillus (FBRA) which is a high fiber brown rice and rice bran mix fermented by the Aspergillus fungus prior to consumption. This intervention failed to find any significant effects, with no significant change to fecal metabolites (total or individual SCFA); total bacterial abundance; and no measurable increase in two target bacterial genus—bifidobacteria and enetrobacteriaecea. However, *in vitro* tests using fecal slurry from 6 of the study participants showed significant increases to both bifidobacteria and SCFAs. One limitation of this study is the use of Terminal Restriction Fragment Length Polymorphism (T-RFLP) to enumerate bacterial species may over simplify diversity due to convergence of species. The author concluded that the status of FBRA as a prebiotic remains unclear. The 2 USA based studies used brown rice flakes providing 11.5 g fiber (17) or 30 g rice bran with 6.3 g fiber (24) to be consumed at any time each day. Both treatments showed significant change to bacterial abundance (Firmicutes, Bifidobacteria, Ruminococcus, Methanobrevibacter, Paraprevotella, Dialister, Anaerostipes, and Barnesiella) compared to baseline, but only rice bran induced increases in SCFA (24)—however given the small sample in this pilot study these results now need replication from larger scale study.

### Impact of Oat Fiber on Gut Microbiota

The outer layers of the oat grain contain a mixture of both insoluble and soluble (beta-glucan) fibers, both of which provide a food source for the gut microbiota. While soluble oat beta-glucans have been established to help lower blood cholesterol levels, little research has been carried out into the effects of oat fiber on the gut microbiota.

Only two studies examined the impact of intact oat fiber on the gut microbiota (25, 26). Conolly et al. (25) examined the effects of consuming a whole grain oat granola vs. a refined grain cereal for breakfast daily in a randomized cross over study for 6 weeks in participants with mild hyperglycemia or hypercholesterolemia. Significant increases in total fecal bacteria, lactobacilli, and bifidobacteria were reported following consumption of wholegrain oat granola, whereas total bacteria and bifidobacteria both fell after consumption of the refined grain cereal. No effects were detected to SCFA. Valeur et al. (26) fed oatmeal porridge (8.5 g fiber) daily for 8 days and also failed to detect change in SCFA levels, however fecal levels of β-galactosidase (lactase enzyme) and urease (protein enzyme) both fell suggesting a rapid adaptation of the microbiota toward utilization of oat fiber.

### Impact of Maize Fiber on Gut Microbiota

Maize has a higher content of starch and a lower bran content compared to other cereal grains, and maize fiber has been less researched compared to other cereal grains.

A single acute intervention study (27) was identified that examined the impact of consuming 48 g of whole grain maize breakfast cereal (14.2 g fiber) on a single occasion on the gut microbiota compared to 48 g of a low fiber maize breakfast cereal (0.8 g fiber). This study was carried out by the same group who demonstrated a prebiotic effect from a breakfast cereal containing wholegrain wheat or wheat bran (11) and extends these findings to wholegrain maize breakfast cereal. As discussed previously use of FISH and specifically selected probes may have limited the range of bacterial change identified. After 3 weeks, significant increases in fecal Bifidobacteria were reported in both the high fiber and low fiber groups, with the greater increase in the higher fiber groups just failing to reach significance (*p* = 0.056), and a non-significant increase in Lactobacillus, Enterococcus, and Atopobium species. Similar to other studies the participants who were most responsive in terms of a bifidogenic effect to the wholegrain cereal had the lowest initial populations of bifidobacteria; conversely, the individuals with the highest initial samples had a less marked response. This study demonstrated a measurable prebiotic effect of a single serve of a wholegrain maize cereal within the context of a freely chosen mixed diet.

### Impact of Mixed Whole Grains on Gut Microbiota

Food based dietary guidelines frequently encouraged an increased consumption of wholegrain cereal foods in order to improve not only fiber intake, but also intake of the wide variety of vitamins, minerals, and antioxidants typically found in the bran layers of cereal grains. Wholegrain cereals include the endosperm, germ, and bran elements of the cereal grain and so have a different nutrient composition to the bran fiber fractions of cereal grains, which could influence effects on the gut microbiota. With no standardized global definition of wholegrain foods comparison of wholegrain intakes, and their associated fiber content can be challenging.

The largest number of studies (*n* = 10, participants = 357) have been carried out into the effects of manipulating intact cereal fiber sources across the whole day, providing wheat fiber as the bulk of the fiber as bread, breakfast cereals, pasta etc., but also permitting some whole oats, rye, and brown rice (15–17, 32–38).

The interventions varied between providing a specific amount of whole grains in the diet: 80 g (32); 105 g (30); 150 g/day (33) or specified amounts of cereal fiber: 13.7 g (31); 21 g (16); 28 g (28, 29); 29 g (5); or 40 g (34, 35). Outcome measurements also varied with 3 studies assessing both fecal bacteria and fermentation metabolites (28, 32, 35), 5 examining only fecal bacteria change (29–31, 33, 34) and the remaining 2 studies measuring only metabolites of fermentation (5, 16).

In terms of outcomes, 9 out of the 10 studies reported significant prebiotic effects from increased consumption of intact cereal fiber, with significant increases also recorded in bacterial diversity (28, 29, 34), Actinobacteria (29), Bifidobacteria (30), Clostridium (33, 40), Lachnospira (29, 34) and non-significant trends to increases in Akkermansia (30) Roseburia (35), Lactobacilli (30), and Enterococcus (33). Significantly decreased levels of pro-inflammatory Enterobacteriaceae were also reported (35). Two studies (29, 34) reported that response to a high fiber intervention is dependent upon the baseline gut microbial richness—those with a limited microbial richness at baseline exhibit a greater microbiota change over time due to dietary fiber increase.

Both Cooper et al. (31) and Ampatzoglou et al. (32) failed to detect significant change in microbiota or fermentation metabolites following increased intake of wholegrain foods. There are a number of potential reasons for this: Cooper et al. included subjects on the basis of self-reported wholegrain intake and fiber intake from others sources was not assessed, in addition the wholegrain foods provided provide just 16% of daily energy intake and so variability in actual food intake, and fiber intake achieved was likely to be high. Microbiota analysis was undertaken on just 28 of the 46 subjects and so the study lacked power to detect anything other than large changes to the microbiota, which coupled with high baseline variability meant that trends were observed, but none were significant. The failure of Ampatzoglou et al. to report significant changes might be down to the use of FISH analysis. FISH relies on selection of probes for target bacterial groups and subsequent research suggests that response to wheat fiber may be greatest in bacteria not targeted by this study. In addition, no account was taken of individual change to microbiota abundance and so larger change for those with lower baseline levels may have been lost in the population averages.

With regard to fermentation metabolites, Vetrani et al. (5) found a significant increase in plasma propionate following consumption of wholegrain foods providing 29 g cereal fiber daily for 12 weeks, and a direct correlation between cereal fiber intake and propionate levels. Individual responses varied with those above the median (responders) showing a reduction in post prandial insulin. No assessment was made of microbiota at baseline or post-intervention and so while we could project that the responders were those with lower levels of target bacteria at baseline we unfortunately do not have the clinical evidence to support this. Similarly, McIntosh et al. (16) measured propionate in feces and also reported a significant increase following 21 g wheat fiber daily for 4 weeks.

### Impact of Reducing Carbohydrate and Fiber Intake on Gut Microbiota

If an increase in fiber intake promotes a bacterial diversity within the gut microbiota, then it would follow that reducing fiber intake would reduce some bacterial phylotypes. This has been demonstrated by 3 research groups who have taken habitually high fiber consumers and reduced their intake of cereal foods and fiber. Lappi et al. (36) replaced rye bread with refined white bread (a 5 g decrease in fiber intake) and measured a significant 37% decline in a specific cluster within the phyla Bacteroidetes. Duncan et al. (37) compared a maintenance diet [28 g Non-Starch Polysaccharides (NSP) fiber] with a medium carbohydrate diet (12 g NSP/day) and a low carbohydrate diet (6 g NSP/day) and found significant reductions in Roseburia, Eubacterium, and Bifidobacteria with each decrement in NSP intake, and concomitant reductions in fecal SCFA, and particularly butyrate. Brinkworth et al. (38) compared a change from a high fiber intake (32 g) to a low fiber diet (13 g). These results also showed significant reductions in Bifidobacteria and both fecal acetate and butyrate with the lower fiber intake.

### Impact of Wheat Bran Arabinoxylans (AXOS) on Gut Microbiota

One of the largest components of wheat bran fiber are arabinoxylans, which can also be consumed as the isolated extract AXOS (arabinoxylan-oligosaccharide). Wheat bran fiber is commonly consumed in foods across the globe, and average intakes of AXOS in the US population have been estimated to be around 7.5 g/day (50). A review of studies examining the effects of consumption of this important wheat bran component on the microbiota was therefore also included.

We identified 9 studies exploring the role of AXOS on the gut microbiota (39–47). Measurements included change to bacterial abundance, breath hydrogen (as a marker of gut fermentation), plasma ferulic acid (ferulic acid is bound to AXOS so increasing plasma levels is a marker of AXOS breakdown by gut bacteria), and SCFA levels. The levels of AXOS provided ranged from 2.2 g (39) through to 18.8 g (46).

Maki et al. (39) showed a dose dependent effect for AXOS, with 4.8 g of AXOS (2 × 2.4 g), contained within two 44 g portions of ready-to-eat-cereal (RTEC) daily, stimulating significantly greater bifidobacteria growth compared to 2.2 g AXOS (2 × 1.1 g) in RTEC, which in turn stimulated greater bifidobacterial growth compared to the control condition of RTEC with no added AXOS. Plasma ferulic acid increased significantly with both 2.2 and 4.8 g AXOS, again with a significant dose dependent relationship. Significant increases in Bifidobacteria were also reported following administration of 2.25 g AXOS (43), two 3.75 g doses of AXOS (41), two 5 g doses of AXOS (44). A single 8 g dose of AXOS gave a 2-fold increase in bifidobacteria (45), and a trend toward increased bifidobacteria (a 1.3-fold increase) was seen following 2.4 g AXOS as a single dose for 3 weeks.

In terms of fermentation metabolites, significant increases in plasma SCFA were recorded in response to 8.9 and 18.9 g AXOS (40), Maki et al. (39) reported no change to acetic acid or propionic acid and a surprising decrease in butyric acid with increasing dose of AXOS, with a significant carry-over for both AXOS treatments suggesting change to microbiota lingered into the 2-week washout periods. The authors suggest this may be due to increased colonocyte activity and uptake of butyric acid stimulated by increasing levels, but that this requires further investigation. Windey et al. (41) found significant effect from 2 × 3.75 g doses of AXOS on fecal and urinary nitrogen, colonic protein fermentation, but no effect on fecal SCFA levels, which may be due to use of a short chain AXOS molecule allowing fermentation in the proximal colon and rapid absorption of any SCFA produced. Change to fecal SCFA were also reported by Francois et al. (45) with total SCFA, acetic, propionic, and butyric acid all significantly increasing following 8 g AXOS for 3 weeks and propionic increasing significantly with the lower intake of 2.4 g AXOS. While most studies focused only on change to limited metabolites, Hamer et al. (47) undertook metabolite fingerprinting following AXOS consumption (10 g/day for 3 weeks) and identified 179 different volatile organic compounds (VOCs) in subject fecal samples, with 24 VOCs present in 70% of participants. The impact of AXOS intake on VOCs was mainly from the reduction of metabolites from protein fermentation, indicating a shift away from protein fermenters and their potentially detrimental metabolites (e.g., phenolic compounds and sulfur containing compounds).

Two of the studies (40, 42) investigated the impact of a single test meal rich in AXOS on fermentation metabolite production the following day after a standardized breakfast meal. Cloetens et al. (42) tested 5 single doses of AXOS (0, 0.2, 0.7, 2.2, and 4.9 g) in 12 healthy participants and measured markers for colonic bacterial metabolism (breath hydrogen measured over 10 h, urine samples collected at 3 time points over 48 h and a stool sample collected at 72 h). A significant increase in both fecal nitrogen (a marker of increased bacterial growth and metabolic activity), and breath hydrogen were observed with AXOS intake of 2.2 and 4.9 g. The second study examined the effect of single higher doses of AXOS (8.9 and 18.4 g) in combination with resistant starch on overnight glucose levels (40), using measurement of SCFA and breath hydrogen as a marker of colonic bacterial fermentation. Significant, dose dependent increases in breath hydrogen, plasma SCFA, (acetate and butyrate) occurred with both AXOS interventions, with no effect from resistant starch. Significant decreases in glucose and insulin responses were also reported with increasing effect from increasing dose of AXOS. A third study gave a higher dose of AXOS (4 × 9.4 g) over a 48 h period (46) and reported significant increases in colonic fermentation on both days 1 and 2 based on hydrogen breath test, with no detrimental effects on gastrointestinal tolerance.

Cloetens et al. further developed their work (43, 44) by demonstrating a significant increase in Bifidobacteria after 2 weeks consumption of 2.25 g AXOS daily, and also established a significant increase in Bifidobacteria and good gastrointestinal tolerance of a high dose of AXOS for 3 weeks (10 g/day), with only a mild increase in flatulence to report. Stimulation of bifidobacteria was most pronounced in participants with the lowest bifidobacteria at baseline, and significant change was not seen at 2 weeks, and only achieved after 3 weeks of consumption (44).

## Discussion

Dietary approaches to manipulate the human gut microbiota have long been used as an approach to improve host health. The aim of probiotic and prebiotic inclusions into the diet are to increase beneficial gut bacteria and their activities, thus generating benefits to human health. These benefits include protection from gastroenteritis by pathogen inhibition, an improved tolerance to lactose, toxins and cholesterol reduction, vitamin synthesis, improved mineral bioavailability, potential protection from bowel cancer, reduced symptoms of irritable bowel syndrome, improved digestion, gut function, and immune regulation (51–53). Recent research highlights a loss of gut microbiota diversity in Western Societies, particularly bacteria belonging to Lactobacillus, Bifidobacterium, Bacteroides, Prevotella, Oxalobacter, and other genera that are essential to our microbial gut community (54). The composition of a “healthy microbiome” has not been precisely defined, and may vary from individual to individual, however evidence suggests that dietary improvements to increasing the amount of fiber, and diversity of foods consumed, to promote microbial diversity could help to maintain health today, and improve health in the future (55).

To date, much of the research into prebiotic effects of dietary fiber has focused on the effects of isolated individual fiber's, and less had been conducted into the potential prebiotic benefits of consuming intact cereal fiber consumed in everyday foods as part of habitual daily diet. However, consumption of a single, highly targeted prebiotic may decrease gut microbiota diversity as bacteria able to utilize that particular energy source “bloom,” changing the overall microbiota composition (29) and conditions within the colon (e.g., a fall on pH due to high levels of SCFA's produced). Assuming microbial diversity to be important in maintenance of good health (55), greater understanding of the impact of provision of multiple, or complex, fiber substrates from the consumption of intact cereal fiber on both species within, and diversity of the gut microbiota is of value. This systematic review is the first to only look at intact cereal fiber sources adding important detail to our understanding of dietary manipulation to promote microbiota diversity.

A summary of the key findings is provided in Table 3. This systematic review provides evidence that increasing daily intake of intact cereal fiber can have a prebiotic effect on gut microbiota composition and activity, helping to support a diverse bacterial population with an increase in bacterial types able to utilize complex fiber structures, with benefits to wide range of bacterial species arising from cross feeding relationships.

Previous authors (8) have found that short term feeding studies failed to support an increase in bacterial diversity, in contrast to observational studies where a habitually high fiber intake supports a more diverse microbiota (56). A recent review (57), supported by intervention study evidence (28) suggests that altering dietary intake over a period of 2–3 days is enough for enriching not only gut microbiota composition with different species, but also overall gut microbiota diversity. Long-term dietary habits may lead to changing states of the gut microbiota diversity, with Prevotella-dominant gut microbiota reported in people consuming a plant-based diet and Bacteroides-dominant gut microbiota in those with higher protein and fat intake (28, 58). However, inter-individual variation in gut microbiota composition before a dietary intervention may also affect responses in terms of both gut metabolites and microbiota composition and must be taken into account.

Individual gut microbiota response to any dietary intervention varies widely depending on starting levels of bacterial species within their established gut microbiota. This was clearly identified in the studies carried out by Carvalho-Wells et al. (27), Martinez et al. (17), and Cloetens et al. (43) who all highlighted that the individuals most responsive to increases in cereal fiber had the lowest starting levels of the target bacteria, with variations in response documented to be as high as 10-fold. Salonen et al. (29) divided their subjects into responders and non-responders to intervention, and identified their non-responders to have high levels of baseline microbiota diversity, implying a link between phylogenetic diversity and ecosystem stability. This suggests that individuals most likely to benefit from an increase in cereal fiber intake are those who habitually consume low fiber cereal foods, those who limit intake of cereal foods (e.g., those following low carbohydrate diets), or those likely to have decreased bacterial diversity, such as older people.

The wide variation in individual response also suggests that this is an exciting area of potential for personalized nutrition interventions. Studies suggest that the gut microbiota could be playing a role in long term conditions such as obesity and Type-2 diabetes (among many others). Identifying the specialist (keystone) bacterial groups for each condition, and cost effective approaches to firstly assess individual microbiota populations; and secondly develop dietary manipulations to support relevant keystone bacterial groups requires further research in order for this to become part of mainstream clinical practice. *In vitro* studies by Duncan et al. (59) have recently identified bacteria from the Lachnospiraceae family (Firmicutes) to be keystone bacteria regarding the utilization of wheat bran. Two studies included in this review also reported significant increase in Lachnospiraceae in response to consumption of wheat fiber (29, 34). As we move beyond isolated fiber supplementation and toward a better understanding of the prebiotic effects of intact dietary fibers our ability to manipulate the gut microbiota through dietary advice will become more targeted, and as a result, more effective.

The short chain fatty acids (SCFA) produced as by-products of bacterial fermentation are difficult to measure due their rapid clearance from plasma. Despite this, several research groups have shown the gut microbiota to be highly responsive, with markers of fermentation stimulated by a single fiber rich meal measurable within the first 24 h following consumption of rye, barley, wheat bran, and wheat bran AXOS (4, 14, 18, 22, 40, 42).

Both beneficial and pathogenic bacteria produce SCFA as a by-product of their fermentation, therefore increases in fecal SCFA may not therefore necessarily be an indicator of benefit. Rahat-Rosenbloom et al. (60) found increased levels of fecal SCFA among overweight individual compared to lean, which was attributed to difference in microbiota populations, with a 5-fold difference in Firmicutes to Bacteriodetes ratio between the overweight and lean individuals. A review of evidence by Lau et al. (61) also supports the role of the Firmicutes:Bacteriodetes relationship in obesity, and a low bacterial diversity leading to unwanted weight gain. Recently, de la Cuesta-Zuluaga et al. (62) demonstrated that in Columbian adults that higher fecal SCFAs are also associated with central obesity, hypertension, subclinical measures of cardiometabolic disease (e.g., inflammation, glycemia, and dyslipidemia), as well as a measure of gut permeability (LPS binding protein). However, microbial diversity showed association with these outcomes in the opposite direction. More research is needed to increase understanding of the relationship between fecal SCFA levels, plasma SCFA, and metabolic health, however evidence to date appears to be suggesting that adopting dietary measures to promote microbiota diversity is likely to be important for long-term health maintenance.

One of the more frequently reported eating occasions for cereal fiber manipulation to take place was the breakfast meal. A simple dietary modification to consume a daily bowl of a high fiber breakfast appears to have a positive impact on the gut microbiota for health adults (11–13, 27), which can be measured within the first 3 weeks (11, 27) and is still maintained after 1 year (13). As little as 5.7 g of wheat fiber was shown to produce significant positive benefits to the gut microbiota, a finding in line with that of So et al. (8) who suggest that <5 g fiber may be sufficient to stimulate bacterial growth. A simple change in eating habits which provides a relatively low, but important boost to fiber intake at a single time point can produce a prebiotic effect within a mixed habitual diet. Other cereal grains reviewed also appear to stimulate gut microbiota at relatively low levels, with as little as 10 g barley fiber, 7 g rye fiber, or 2.2 g AXOS providing measurable significant effect. Breakfast is often shown to provide a significant contribution to daily fiber intakes of western populations, and is an occasion where switching to higher fiber foods is more easily accepted by the consumer. Whether delivering a single bolus of fiber at breakfast has different stimulatory effects on the gut microbiota compared to delivery of a steady stream of fiber throughout the day is a potential area for future research.

One important finding for intact cereal fiber consumption is the support of a diverse bacterial community, and the more complex fibers, such as wheat bran appear best placed to promote this diversity. Bacterial diversity is known to vary depending on habitual diet consumption, with communities living in agrarian societies and those consuming diet with high levels of plant based foods, such as vegans and vegetarians possessing a higher level of bacterial diversity compared to communities with omnivorous dietary intakes (53, 56, 63–65). However, change to bacterial diversity was only measured in two of the studies reported here (17, 29).

One further consideration when comparing the impact of fiber sources on the gut microbiota is not only the cereal source of the fiber, and its complexity, but also the preparation and processing of the grain in question. Cereal grains differ in composition, with varying amounts of total dietary fiber, insoluble fibers and soluble fibers, and processing (e.g., milling, heating, flaking, or extrusion) of grains has been found to affect *in vitro* fermentation differently depending on the grain (66). Grain fibers are not all equal in their potential for prebiotic effect and the varying effects of preparation and processing of each grain complicates this further.

Of the 40 studies included into this review, 25 reported change to bacterial levels (either in terms of bacterial abundance at genus or species level or population diversity), and 23 out of these 25 studies reported a prebiotic outcomes. In terms of fermentation metabolites 26 studies reported on these with 25 showing increased levels of fermentation. Two studies showed no effect of cereal fiber consumption on gut microbiota composition, which can be explained in part by methodological weaknesses. No studies showed any negative implications from consuming increased levels of intact cereal fibers and their sub fractions in the metabolic parameters measured (typically digestive comfort, bowel movements, weight change, blood lipid, and glucose responses etc.), aside from occasional mild and transient increases in flatulence.

The studies reviewed here provide some key information and learnings for future research. Several *in vitro* studies have shown that not all Bifidobacterium species can degrade the wheat bran arabinoxylans, reinforcing the need for accurate identification of bacteria studied. For example, *in vitro* fermentation models have found that, arabinoxylans are completely degraded by *Bifidobacterium adolescentis* and Bacteroides vulgatus, partially degraded by *Bifidobacterium longum* and *Bacteroides ovatus*, and not degraded by *Bifidobacterium breve* and *Bifidobacterium infantis* (67). Cloetens et al. (44) and Windey et al. (41) both reported on *Bifidobacterium adolescentis* counts and have corroborated the *in vitro* observations, that intake of AXOS by the healthy human participants stimulated *Bifidobacterium adolescentis*. It should be noted that Bifidobacteria do not produce butyric acid, and studies reporting an increases in Bifidobacteria accompanied by increases in butyric acid levels (40, 45) therefore suggest a mechanism of cross-feeding with acetate- or lactate converting bacteria may be involved in increased colonic butyric acid production. Subsequent work has confirmed that the ability utilize arabinoxylans is strain specific within the Bifidobacterium species. For example *B. longum* subsp. longum LMG 11047, and *B. longum* subsp. longum CUETM 193 are both able to fully breakdown complex AXOS molecules, whereas *B. longum* subsp. longum NCC2705 is only able to utilize free arabinose and not more complex AXOS (68). Specificity not only in terms of bacterial species, but also in terms of strain is likely to gain precedence as next generation sequencing techniques become more accessible.

Wheat bran formed a particular interest within this systematic review due to its contribution to the fiber intake of Western populations. A number of research papers reviewed here suggest that wheat bran is a complex fiber, supporting the growth of distinct specific bacterial populations (14, 16, 43). Research has shown that bran particles are colonized by bacteria after 24 h (no earlier time period was measured) (67), and with normal colon transit times reported at around 70 h (69). Allowing sufficient follow-up time for complex cross-feeding relationships to develop and stabilize is an important learning for research going forwards. Wheat bran has been ingeniously described as consisting of a unique combination of fermentable and non-fermentable fibers, entangled in a porous insoluble network that could serve as an ideal “dinner table” for micro-organisms (70). Cellulose and highly branched arabinoxylans are resistant to fermentation and are proposed to act as a physical surface or “table” onto which bacteria attach. Fermentable substances in the wheat bran particle (such as starch, proteins and less substituted arabinoxylans) then provide the “dinner” for these attached microbes. This concept of a unique microbiota attaching to wheat bran particles has been explored *in vitro* using fecal samples from healthy donors (70). Wheat bran particles were found to host a distinct microbial community, compared with the luminal environment. The concept of separate bacterial colonies co-existing to fully utilize the particulate and luminal environments of the human colon could partly explain the variation in response to different fiber types in human feeding studies.

This to our knowledge is the first review looking solely at the influence on gut microbiota arising from the consumption of intact cereal fibers. Comparison of outcomes from this and other systematic reviews of the prebiotic potential of isolated fibers is limited by the lack of overlap.

Kellow et al. (7) reviewed the impact of dietary prebiotic supplementation (e.g., fructans, oligosaccharides, or inulin) on parameters associated with the development of metabolic abnormalities such as obesity, glucose intolerance, dyslipidaemia, non-alcoholic fatty liver disease, and low grade chronic inflammation. The review found convincing evidence from short-term high-quality human trials to support the use of dietary prebiotics as a potential therapeutic intervention for the regulation of appetite and the reduction of circulating postprandial glucose and insulin concentrations, however the shift in microbiota responsible for these benefits was not elucidated.

The recent review by So et al. (8) included only Randomized Controlled Trials with either a placebo, low fiber diet or habitual diet group as comparators. Two outcomes were reported—diversity and richness of the microbiota population between groups and change to specified groups of bacteria. Of the 64 included studies, 52 involved supplementation with a prebiotic or candidate prebiotic fiber and just 12 examined intervention using whole foods with intact fibers. Prebiotic fiber supplementation increased specified target bacteria such as *Bifidobacterium* species and *Lactobacillus* species, with as little as 5 g of fiber sufficient to significantly increase *Bifidobacterium* species. The considerable degree of heterogeneity in microbiota between participants was noted for all analysis sub-groups. Only a small number of studies reported effect on microbiota diversity and the overall lack of apparent effect on diversity was noted. Long-term dietary diversity as opposed to changes in isolated nutrients or foods over a short period of time may be a stronger driver of microbial diversity. The review authors made an interesting observation that microbial diversity was not compromised by any of the reported interventions, which helps to support the case for favorable effects of dietary fiber on the gut microbiota.

Sawicki et al. (71) took an Evidence Mapping approach to explore the influence of dietary fiber on the human gut microbiota. This mapping exercise highlighted that much of the current literature has shown positive effects of dietary fiber on gut function or beneficial bacterial species, or positive effects of dietary fiber on specific health outcomes, but few seem to be directly measuring these outcomes together, to provide evidence of a dietary fiber-modulated gut microbiota and health outcome.

### Methodological Issues

One key limitation is the number of studies measuring change in plasma or fecal SCFA's. *In vivo* measurements of SCFA's in plasma or feces do not reflect levels reaching the liver or the colon walls as they are rapidly taken up and utilized by both sites. Changes measured in either plasma or feces are therefore likely to provide a gross underestimate of actual levels of change.

Characterization and measurement of change in the gut microbiota provides challenges for research. Culture-based methods of measurement underestimate bacterial diversity, and as many bacteria cannot been grown in culture these are lost to measurement using these techniques. Culture-based methods have been largely superseded over the past decade by culture-independent methods based on the characterization of 16S ribosomal ribonucleic acid (rRNA) genes, however while much improved, 16S rRNA gene sequencing is often limited to identifying bacteria at genus level, and changes at species, and strain, level will often not be reported. Using the example of Bifidobacterium previously explained above, there will be a prebiotic effect for some strains following cereal fiber consumption while other strains will not demonstrate a prebiotic effect—there could be significant compositional change but no detectable change in total *Bifidobacterium* abundance which could lead to conclusion of ‘no effect. In addition, 16S rRNA analysis depends on a database of reference genes: thus the assignment of both genus and species may be affected according to the reference database selected (72).

A large amount of human bacteria still remain to be characterized, although advances in next-generation sequencing and metagenomics are rapidly expanding knowledge of the number and diversity of human bacteria. Many studies target specific reference bacteria (most commonly bifidobacteria or lactobacilli), which is likely to underestimate the benefits arising from fiber intake. Metabolic response of bacteria varies widely at species level as shown by Van Laere et al. (73) with *Bifidobacterium adolescentis* and *longum* able to ferment wheat bran arabinoxylans compared to *Bifidobacterium breve* and *infantis* who are unable to utilize the complex structure of wheat bran fiber. Examination of the gut microbiome both at species level and with regard to metabolic response is needed to further expand our knowledge of the effects of intact fibers on microbiota composition and diversity.

It is estimated that each individual carries an estimated 160 bacterial species (3), and studies reported here highlighted the wide diversity in species identified in the fecal samples of participants (4, 5, 17, 30, 43). Several studies identified that individuals with low levels of specific target bacteria had large responses to an intervention compared to those with high starting levels for whom population increase was more limited (17, 27, 43). Failure to consider habitual fiber intake, habitual food diversity, and the composition of gut microbiota at individual level within a study population could mean that a substantial change in some subjects be masked by a more negligible total population response due to low responders within the population sample.

Recent work has indicated that breakdown of complex cereal fiber structures by gut bacteria is dependent on the presence of carbohydrate active enzymes (CAZ-enzymes) able to cleave the bonds between sugar molecules forming the backbone of fiber molecules and other bioactive compounds (74). While humans are thought to possess just 17 different CAZ-enzyme families, limiting us to digestion of relatively simple carbohydrate molecules, the gut microbiota is thought to possess over 170 different CAZ-enzyme families (74). Some bacteria have a small range of CAZ-enzyme limiting their utilization to relatively few carbohydrates and so are termed “specialists,” whereas others have a wider array of CAZ-enzymes, are able to utilize a large number of different carbohydrate structures and are termed “generalists.” As research continues to develop, consideration should be given to the specificity of the range and types of bacterial species measured in order to capture the most appropriate bacterial responders for the fiber type/s under examination.

While the gut bacteria respond rapidly to provision of fiber substrates, cross-feeding relationships are complex and take time to establish and stabilize. The ideal duration of intervention to allow a diverse and stabile gut microbiota to establish is not yet known, however is likely to extend beyond the 2–3 weeks studied by much of the work published to date. The full potential benefits that could arise from providing the gut microbiota with a diverse, high fiber diet every day have therefore yet to be accurately established and longer term trials of several months in length are needed.

Many studies controlled for confounders (e.g., use of antibiotic, probiotics of gastrointestinal disease) in the statistical analysis, via inclusion of many covariates in the analysis. Whilst this is an important approach for controlling for confounders, the benefit of including many covariates into a statistical model should be balanced with the issue of overfitting, particularly in those studies with small sample sizes. Therefore, there is a need for studies with larger sample sizes and careful selection and included covariates.

Habitual diet, environmental factors, geographical location, and race all influence gut microbiota composition. All of the studies included in this review included adults from a single geographical locale, participants were predominantly Caucasian, with a single study conducted in Japan. Given the known diversity of microbial composition between individuals it cannot be assumed that results found in any or all of these studies will transfer across regions or between different ethnicity.

### Research Recommendations

Although knowledge has advance substantially in recent years, much remains to be discovered regarding the gut microbiome and how to achieve the greatest potential benefits from dietary manipulations. The wide variation in individuals microbiota highlighted in a number of studies reported here requires further research: what is the level of variation between individuals and what are the factors contributing to this? Can dietary manipulation of fiber type correct microbiota dysbiosis and over what time frame? Response to dietary intervention appeared to stimulate little effect in a sub-group of people lacking key bacterial species, however this was measured over days or weeks rather than months. Long-term studies are needed to establish whether maintaining a high fiber intake could overcome initial shortfalls in the gut microbiota population. Could development of cost-effective and reliable mechanisms to elucidate an individual's microbiome hold the potential to open up a new and exciting field of personalized targeted nutrition recommendations to promote microbiota health? This too needs to be explored.

Research results in the past may have been limited by measurement of bacterial species which are not specialist fermenters of the fiber substrate provided. This may be particularly relevant for wheat bran where key, highly specialized bacterial groups have now been identified. It may be both relevant and appropriate to repeat previous studies attempting to elucidate the potential prebiotic effect of wheat bran fiber, with more participants over a longer follow up period, to understand more clearly the potential benefit (or otherwise) of increasing wheat bran fiber intake to our gut microbiota.

## Conclusions

The colonic microbiota community must typically be in a state of continuous change over time, driven by short-term changes in dietary intake. This review supports a role of intact cereal fibers in promoting gut microbiota diversity and abundance. The strongest evidence lies in the role of wheat bran and wholegrain wheat fiber promoting gut microbiota diversity, as this is the cereal fiber which demonstrated the most consistent prebiotic effects on gut microbiota composition both in its intact form within commonly consumed foods, and in terms of its key active constituent AXOS, with demonstrable effects arising from increases in wheat fiber as low as 6 g/day. Individual response to fiber intervention varied in terms of microbiota response, however several studies concur that those with the greatest response were those with the lowest initial target bacterial levels. Those with the lowest fiber intakes therefore potentially have the most to gain from increasing fiber intake. Moving forwards it is important that future studies take account of individual variance in response and the species within the microbiota responsible for this to further understanding of potential to personalize dietary recommendations of fiber intake to fit gut microbiota profile.

With few notable negative side effects reported from increasing intake of cereal fiber, and evidence accumulating for a wide array of beneficial health benefits to be gained from gut microbiota composition, increasing population fiber intakes remains a key public health goal. As knowledge grows of our symbiotic relationship with our gut microbes, so does knowledge of what helps the microbiota composition to increase in abundance and/or diversity, which at its simplest is to eat plenty of dietary fiber. Compared to recommended, dietary intake of fiber remains universally low in Western societies (75). Continued encouragement of simple dietary changes to increase intake of intact cereal fibers (for example to choose breakfast cereal rich in wheat bran, wholegrain wheat or rye breads, and brown rice), should remain a key focus of dietary advice provided at individual, community, and at national levels.

## Author Contributions

AJ and KA developed the study premise. Abstraction of data from articles was undertaken by AJ. AJ developed the initial draft of the paper and both authors contributed equally to, and approved the final version of the manuscript.

### Conflict of Interest Statement

The authors declare that the research was conducted in the absence of any commercial or financial relationships that could be construed as a potential conflict of interest.
